# High Dose Rate versus Low Dose Rate Brachytherapy for Oral Cancer – A Meta-Analysis of Clinical Trials

**DOI:** 10.1371/journal.pone.0065423

**Published:** 2013-06-10

**Authors:** Zhenxing Liu, Shengyun Huang, Dongsheng Zhang

**Affiliations:** Department of Oral and Maxillofacial Surgery, Provincial Hospital Affiliated to Shandong University, Jinan, China; Northwestern University Feinberg School of Medicine, United States of America

## Abstract

**Objective:**

To compare the efficacy and safety of high dose rate (HDR) and low dose rate (LDR) brachytherapy in treating early-stage oral cancer.

**Data Sources:**

A systematic search of MEDLINE, EMBASE and Cochrane Library databases, restricted to English language up to June 1, 2012, was performed to identify potentially relevant studies.

**Study Selection:**

Only randomized controlled trials (RCT) and controlled trials that compared HDR to LDR brachytherapy in treatment of early-stage oral cancer (stages I, II and III) were of interest.

**Data Extraction and Synthesis:**

Two investigators independently extracted data from retrieved studies and controversies were solved by discussion. Meta-analysis was performed using RevMan 5.1. One RCT and five controlled trials (607 patients: 447 for LDR and 160 for HDR) met the inclusion criteria. The odds ratio showed no statistically significant difference between LDR group and HDR group in terms of local recurrence (OR = 1.12, CI 95% 0.62–2.01), overall mortality (OR = 1.01, CI 95% 0.61–1.66) and Grade 3/4 complications (OR = 0.86, CI 95% 0.52–1.42).

**Conclusions:**

This meta-analysis indicated that HDR brachytherapy was a comparable alternative to LDR brachytherapy in treatment of oral cancer. HDR brachytherapy might become a routine choice for early-stage oral cancer in the future.

## Introduction

Oral cancer is posing an ever increasing threat to global health. The annual case number is estimated around 275,000, two-thirds of which occur in developing countries [1], [2]. The current four main treatment modalities for oral cancer are surgery alone, radiotherapy alone, surgery with radiotherapy, and chemotherapy with or without surgery and radiotherapy [3]. With the improvement of treatment modalities over decades, the quality of life comes to our attention.

Owing to its excellent local control rates, acceptable side effects, and high quality of life, brachytherapy has been demonstrated as a sole treatment modality or a supplementary method for oral cancer. It provides a high localized dose of radiation, with rapid fall-off and short overall treatment duration [4]. Low dose rate (LDR) brachytherapy has been reported with a superior outcome in treatment of carcinoma of the lip, tongue, floor of mouth, oral mucosa, base of tongue, buccal mucosa, soft palate, etc. Nowadays, LDR brachytherapy is challenged by high dose rate (HDR) brachytherapy, as the latter shows advantages in avoiding radiation exposure to medical personnel and shortening the treatment period. As a result, HDR brachytherapy has become widely accepted in treating carcinoma, especially in gynecological cancer, breast cancer, and prostate cancer [5]. However, the efficacy and safety of HDR brachytherapy, compared with LDR brachytherapy, remains controversial.

Thus, we performed this meta-analysis to evaluate the efficacy and safety of HDR versus LDR brachytherapy in treatment of oral cancer in terms of local recurrence, overall mortality, and treatment complications.

## Methods

### Literature Search and Study Selection

A systematic search of MEDLINE, EMBASE and Cochrane Library databases, restricted to English language up to June 1, 2012, was performed to identify potentially relevant studies. Analysis methods and inclusion criteria were specified in advance and documented in a protocol. The literature search strategy is presented in Table 1, and relevant hits are 211 (for MEDLINE and EMBASE) and 114 (for Cochrane Library).

**Table 1:** Literature search strategies performed.

	Search term entry
#1	cancer or carcinoma or SCC or CA or tumor or tumour or malignan* or neoplas* or HNSCC or OSCC
#2	‘head and neck’ or ‘oral cavity’ or mouth or oral or intra-oral or gingiva* or lip* or tongue or palatal or palate or gum* or buccal
#3	#1 AND #2
#4	‘brachytherapy’ or ‘brachytherapy implant’ or ‘brachytherapy device’ or ‘interstitial radiotherapy’ or ‘implant radiotherapy’ and (‘low dose rate’ or ‘low dose’ or low or LDR) and (‘high dose rate’ or ‘high dose’ or high or HDR)
#5	#3 AND #4
#6	outcome or ‘follow up’ or ‘local control rate’ or LCR or ‘local recurrence’ or ‘local failure’ or relapse or survi* or complication or ‘adverse reaction’ or ‘adverse effects’ or toxic or toxicity or necrosis or nucler or mucositis
#7	#5 AND #6

SCC: squamous cell carcinoma, CA: carcinoma, HNSCC: head and neck squamous cell carcinoma, OSCC: oral squamous cell carcinoma, HDR: high dose rate, LDR: low dose rate, LCR: local control rate.

**Figure 1:**
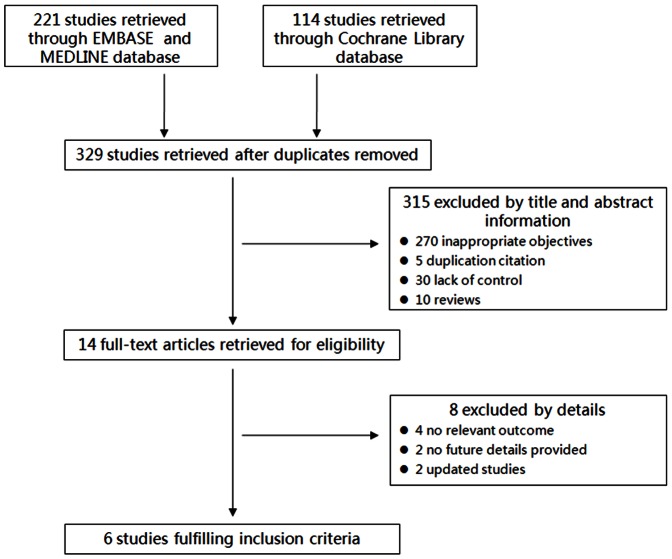
PRISMA flow chart of the meta-analysis.

The inclusion criteria for this review were: (a) randomized controlled trials (RCT) or non-randomized controlled trials; (b) trials recruiting tumors of the oral cavity, including tongue, buccal mucosa, palate, gingival, and cancers of the lip; (c) LDR brachytherapy and HDR brachytherapy were applied and compared in the therapy; (d) local control rate, overall survival and treatment complications were described as reference standard; (e) trials with a minimum follow-up of 18 months; (f) at least 25 patients were included. Two investigators (Z.X.L. and S.Y.H.) independently judged study eligibility. Disagreements were resolved by discussion. Literature selection was present in the PRISMA flow chart (Figure 1) according to the PRISMA guidelines [6], [7].

### Data Extraction

Two investigators (Z.X.L. and S.Y.H.) extracted data from retrieved studies independently and resolved controversies by discussion. Study characteristics that were collected included (a) first name, (b) year of publication, (c) the anatomical location of the tumor, (d) brief description of literature (number of patients, implant species, dosage of brachytherapy, local control rate, overall survival and treatment complications), (e) length of follow-up, and (f) quality of trials.

### Statistical Analysis

The outcome data of local recurrence, overall mortality and treatment complications was summarized. OR and 95% CI (confidence intervals) were employed to evaluate each outcome. Heterogeneity across the trails was evaluated by I^2^ statistics, which describes the percentage of total variation across studies that is due to heterogeneity rather than chance [8]. Odds ratio (OR) was calculated using fixed effect model or random effect model (Mantel-Haenszel method) [9] according to I^2^ values (low: 0–25%; moderate: 25%–50%; high: 50%–75%; extreme: 75%–100%) [10]. To ascertain the validity of included studies we conducted the meta-analysis according to the Cochrane risk of bias tool and Newcastle-Ottawa Scale (NOS) [11], [12]. The potential publication bias was assessed using “funnel plot”. The GRADE approach was used to present the overall quality of evidence as recommended by the Cochrane Handbook for Systematic Reviews of Interventions [11]. GRADEprofiler software (version 3.6) was used to prepare the ‘summary of finding’ table. All the analyses (including sensitivity analyses) were performed using RevMan 5.1.

## Results

**Figure 2:**
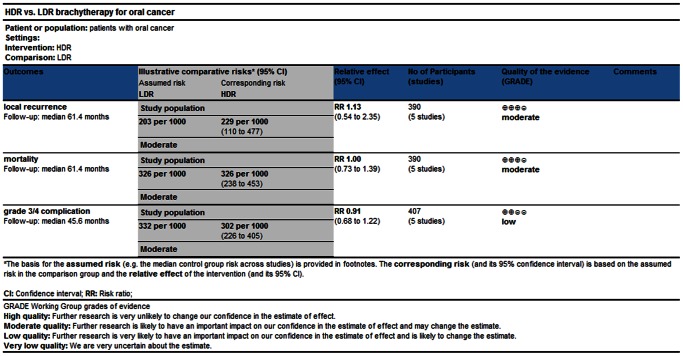
Summary of findings table using GRADE methodology.

We included 6 trials in total, consisting of one RCT and five trials with control. Of those eligible trials, 607 patients treated with LDR (n = 447) or HDR (n = 160) were taken into account. Four studies compared LDR versus HDR brachytherapy alone, while two others combined adjuvant routine radiotherapy. The RCT showed a low risk of bias according to the Cochrane Collaboration’s tool for assessing risk of bias [11]. Five controlled trials presented the Newcastle-Ottawa Scale (NOS) of 6–8. The retrieved studies are present in table 2 [13]–[18], and the levels of evidence were also described referring to Oxford Centre for Evidence-based Medicine [19]. The study quality was also estimated following the GRADE system, and figure 2 shows the outcomes of ‘summary of finding’. It was moderate for local recurrence and mortality and low for Grade 3/4 complications.

**Table 2:** Characteristics of literatures investigating LDR vs. HDR for oral cancer.

Author	Year	Anatomy	LOE	LDR			HDR		
				Pts	Implants	Dose(Gy)	Pts	Implants	Dose(Gy)
Ghadjar^13^	2011	Lip	2b	70	^192^Ir	60	33	^192^Ir	36
Arrate^14^	2010	Lip	2b	100	^192^Ir	60–70	21	^192^Ir	45–50
Umeda^15^	2005	Tongue	2b	78	^226^Rd/^137^Cs	61	26	^192^Ir	59
Kakimoto^16^	2003	Tongue	2b	61	^192^Ir	69	14	^192^Ir	49
Inoue^17^	2001	Tongue	1b	26	^192^Ir	70	25	^192^Ir	60
Inoue^18^	1997	Floor of mouth	2b	41	^198^Au	72	16	^192^Ir	53

LOE: levels of evidence, Pts: patients.

### Local Recurrence

**Figure 3:**
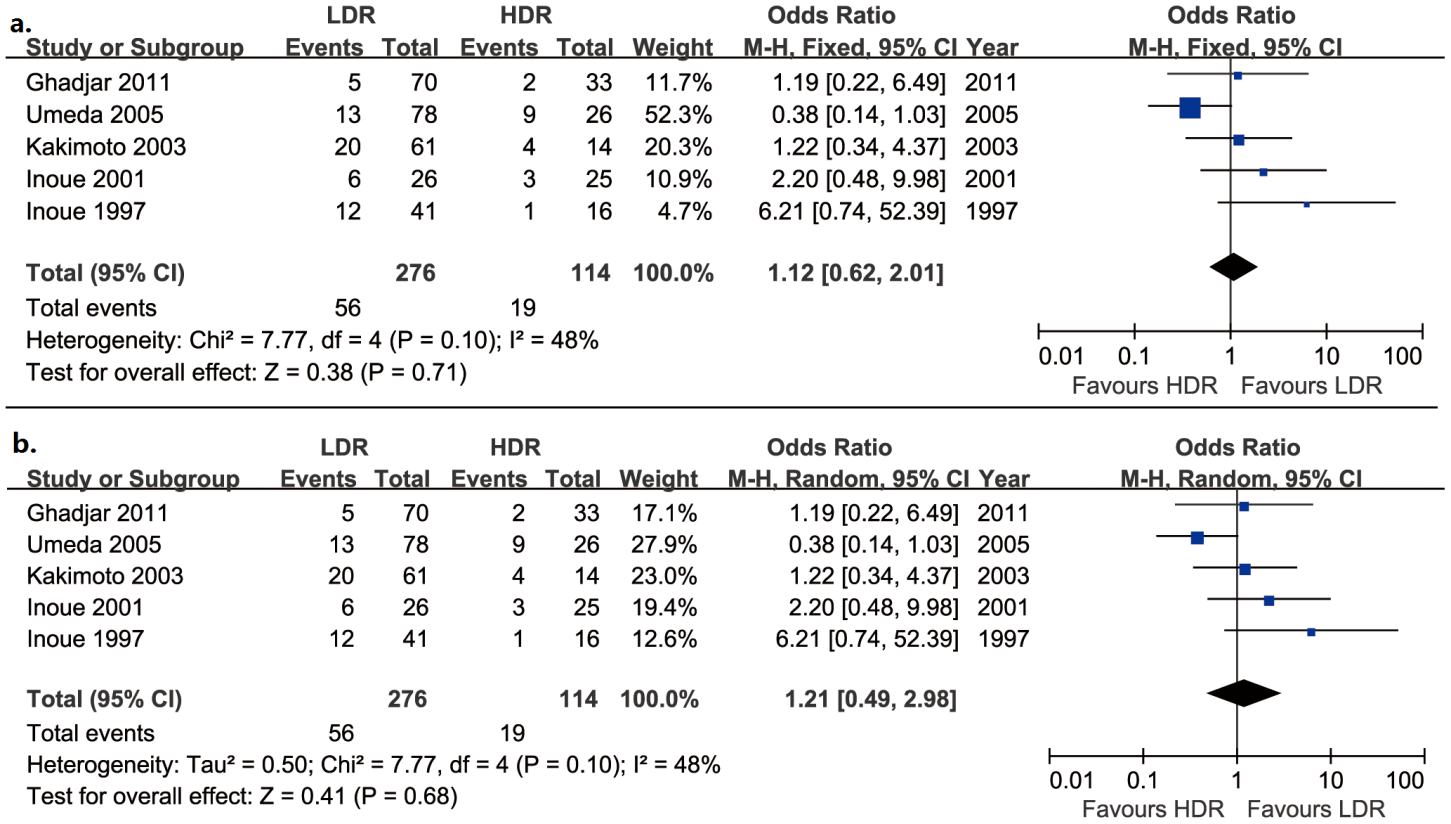
Local recurrence of oral cancer treated with LDR vs. HDR brachytherapy (a. fixed effects model; b. random effects model).

Five trials were suitable for inclusion to analyze of the local control rate, which included 276 patients in the LDR brachytherapy group and 114 patients in the HDR brachytherapy group. Heterogeneity across these trails was moderate (I^2^ = 48%), and fixed effect meta-analysis model was used. There was no statistically significant difference in local recurrence between LDR brachytherapy group and HDR brachytherapy group (OR = 1.12, CI 95% 0.62–2.01), as Figure 3a.

The effect model was changed into random effect model to perform sensitivity analysis, and the difference in local recurrence between LDR group and HDR group was still not statistically significant. The result under the random effect model (figure 3b) was in accordance with that under the fixed effect model.

### Overall Mortality

**Figure 4:**
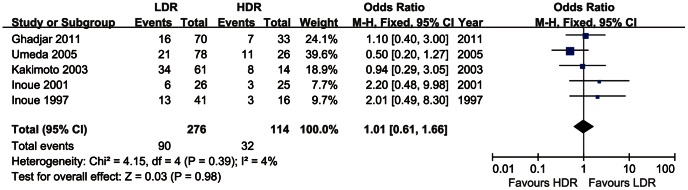
Overall mortality of oral cancer treated with LDR vs.HDR brachytherapy.

Overall survival (OS) was reported in five trials as one outcome indicators. 390 patients were involved to this meta-analysis. The result implied a slightly higher mortality for LDR brachytherapy (90/276 = 32.6%) versus HDR brachytherapy (32/114 = 28.1%). However, the odds ratio (OR = 1.01, CI 95% 0.61–1.66) showed no statistically significant difference between two groups. The fixed effect model was applied, as the heterogeneity was low (I^2^ = 4%). Of all the trials, the difference of overall mortality between LDR brachytherapy group and HDR brachytherapy group was not statistically significant, as figure 4.

### Grade 3 and Grade 4 Complications

**Figure 5:**
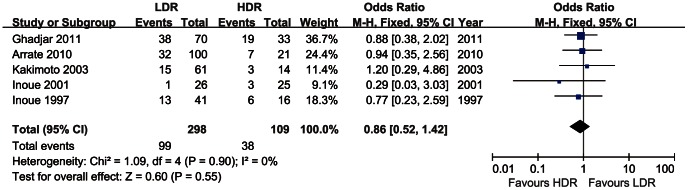
Grade 3 or Grade 4 complications of oral cancer treated with LDR vs. HDR brachytherapy.

Five trials including 407 patients provided information on the Grade 3 and Grade 4 complications of oral mucosa after LDR/HDR brachytherapy. The odds ratio, expressed as LDR group versus HDR group, was 0.86 (CI 95% 0.52–1.42). There was a low heterogeneity among the trials (I^2^ = 0%), and the fixed effect model was used, as figure 5.

## Discussion

It was estimated that 128,000 people worldwide died from oral cavity cancer (including lip cancer) in 2008 [1]. The mortality of oral cancer varies geographically, about 3–4 per 100,000 for men and 1.5–2.0 per 100,000 for women for most countries [2]. The five-year survival rate for cancers of the tongue and oral cavity is around 50% while over 90% for cancer of the lip [2]. Radiotherapy can be chosen as the sole treatment for T1-2N0 oral cancer according to the NCCN clinical practice guidelines for head and neck cancer (version 2, 2011) [20], avoiding potential severe masticatory, speech, and swallowing disturbances resulted from surgery. Radiotherapy for oral cancer consists of teletherapy and brachytherapy. Brachytherapy showed good efficacy over early cancers of the head and neck [21] and lower damage to normal surrounding tissues, and has become an important therapeutic alternative to conventional radiotherapy for oral cancer in Japan [22].

LDR and HDR are two main types of brachytherapy for oral cancer, and the former has come into widespread use and become the gold standard in brachytherapy [23]. Meanwhile, HDR brachytherapy has become more and more popular during the last 3 decades, owing to the development of remote after-loading technology at the aspect of high-intensity radioactive sources, treatment planning software, and sophisticated computerized remote after-loading devices [24]. HDR brachytherapy offers numerous advantages over LDR brachytherapy, including (i) accurate calculations made possible by complete fixation of the guide tubes, (ii) parallel source arrangement with sophisticated technique, (iii) homogeneous dose distribution due to stepping source optimization, (iv) better patient care in normal wards without medical staff radiation exposure, administration on an outpatient basis in several cases [22], and (v) no shifting of seeds within the tissues. However, it is still controversial whether HDR brachytherapy can replace LDR brachytherapy in treating oral cancer, in terms of efficacy and safety [14], [17], [25], [26].

The results of this meta-analysis show that HDR brachytherapy is an alternative to LDR brachytherapy in treating early-stage oral cancer. The local recurrence and overall mortality are similar in both groups for early-stage or local advanced oral cancer. (Figure 2, 3 and 4).

Brachytherapy is mainly applied as a sole treatment for early-stage oral cancer without regional metastasis, and demonstrates excellent cure rate [27]. Most studies that involved HDR brachytherapy suggested a similar or even better outcome in local control rate and overall survival when compared with LDR brachytherapy. However, some trials held the opposite view [28]. This divergence was probably caused by various technical factors, such as delineation of gross tumor volume (GTV), treatment planning software, remote after-loading devices, and quality of radioactive sources. These indicators suggest that HDR brachytherapy should be applied with perfect technical support and caution.

Sometimes, brachytherapy is applied to early-stage oral cancer treatment in combination with external beam radiotherapy, and presents satisfactory outcome. At the same time, HDR brachytherapy presents better performance than LDR brachytherapy. Inoue et al. [18] used interstitial radiotherapy ± EBRT for carcinoma of the floor of the mouth. The local control rates were 62% for LDR+EBRT group and 90% for HDR+EBRT group, while were 71% for LDR group and 94% for HDR group. Guinot et al. [29] reviewed data form 50 patients treated for oral tongue carcinoma with HDR-ISBT ± EBRT. Exclusive HDR cases showed LC in 100% of the cases, and the combined group (EBRT+HDR) showed LC in 80%. These studies suggested that HDR brachytherapy can be an alternative to EBRT for early-stage oral cancer.

The most common complication is mucosal mucositis or/and necrosis during or/and after brachytherapy, and bone necrosis can be observed in some cases. Our results showed that there was no statistically significant difference between LDR group and HDR group in complications. Similar Grade 3 and Grade 4 complications were presented in both groups. (Figure 5) However, in theory, the rate of complications might rise up along with the increase of dose rate. The results indicated that the HDR brachytherapy fractionation schedule was balanced out by the continuous LDR brachytherapy.

Many attempts have been carried out to seek the optimal HDR brachytherapy protocol [30]–[32]. So far, the protocols vary greatly among different institutes. In this meta-analysis, HDR brachytherapy protocols showed a range of 30–60 Gy/6–13 Fr/5–7 d [13]–[16], [18], and were practiced upon previous experience.

Generally, a reasonable HDR brachytherapy schedule is mainly composed of the following parts: (i) patient selection, (ii) pre-treatment preparation, (iii) treatment strategy, (iv) treatment execution, and (v) post-treatment care and follow-up. At the same time, competent imaging plays a crucial role in the treatment strategy, especially in treatment planning system and remote after-loading system. A better imaging can improve the localization of tumor and distribution of brachytherapy dose [33]. Three dimensional (3D) imaging, such as CT and/or MRI, has been widely used in brachytherapy for uterine cervical cancer [34], [35]. More efforts should be directed toward treating oral cancer with 3D imaging in the future.

Meanwhile, the results of this meta-analysis should be interpreted with caution, as the pooled data suffered risk of bias in four areas: selective reporting, location of studies, incomplete outcome and blinding of outcome assessment.

### Conclusions

To sum up, we performed this meta-analysis of HDR versus LDR brachytherapy for oral cancer in terms of local recurrence, overall survival, and complications in data pooled from one RCT and five controlled trials. The results showed HDR brachytherapy was a competent alternative to LDR brachytherapy for early-stage oral cancer. More clinical trials are needed to explore the optimal HDR brachytherapy protocol.
